# Effect of Weather Aging on Viscoelasticity and Fatigue Performance of Asphalt Mastic

**DOI:** 10.3390/ma14206163

**Published:** 2021-10-18

**Authors:** Gang Xu, Yixin Zhou, Yu Zhu, Rui Wang, Xianhua Chen

**Affiliations:** 1 National Demonstration Center for Experimental Road and Traffic Engineering Education, School of Transportation, Southeast University, 2 Sipailou, Nanjing 210096, China; 230198682@seu.edu.cn (G.X.); 220203363@seu.edu.cn (Y.Z.); 220183096@seu.edu.cn (R.W.); 2 Jiangsu Expressway Management Center, Nanjing 211189, China; zhuyuJS@126.com

**Keywords:** asphalt mastic, weather aging, viscoelasticity, fatigue performance

## Abstract

The long-term effect of climate factors, such as sunlight, oxygen, and water, leads to the performance degradation of the asphalt mastic, which is the binding part in the asphalt mixture. It is not conducive to satisfy the long-term performance requirements of long-life asphalt pavement. In this study, five kinds of base asphalt mastic and styrene-butadiene-styrene (SBS) modified asphalt mastic were prepared with the filler-asphalt ratio of 0.6, 0.8, 1.0, 1.2, and 1.4. The indoor simulated weather aging tests were carried out considering multi-factors including sunlight, oxygen, and water. The master curves of the complex shear modulus and phase angle of the asphalt mastic with different aging degrees were obtained by the frequency sweep test. The curves of fatigue damage characteristics and fatigue life were fitted based on the viscoelastic continuum damage (VECD) model. The influence of weather aging on the viscoelasticity and fatigue performance of asphalt mastic were analyzed. Results indicated that the effect of weather aging increases the elastic component and decreases the viscous component. The fatigue performance of SBS modified asphalt mastic was better than that of base asphalt mastic. As the aging degree deepens, the brittle failure characteristics of asphalt mastic with a higher filler–asphalt ratio were more obvious. The base asphalt mastic becomes more sensitive to the strain level due to weather aging, and its fatigue life increased under the low strain loading and decreased under the high strain loading. The fatigue performance of SBS modified asphalt mastic was less sensitive to the strain level. The fatigue life reduced after aging under low and high strain load. Taking the impact of weather aging on the fatigue performance into consideration, the optimal filler–asphalt ratios of the base asphalt mastic SBS modified asphalt mastic are 1.0 and 1.2, respectively.

## 1. Introduction

For long-life asphalt pavement with a service life of 40 to 60 years, the long-term effect of the weather aging will gradually lead to the performance degradation of the asphalt mastic, which is the binding part in the asphalt pavement. It will cause irreversible performance degradation and reduce the service life. Therefore, it is necessary to study the influence of aging on the long-term performance of asphalt mastic [1]. During the service of asphalt pavement, many factors could lead to the aging of asphalt mastic, such as sunlight, oxygen, and moisture. These factors react with the asphalt mastic and then resulting in aging, which include thermal oxidative aging, photo-oxidative aging, and water-damage aging.

For thermo-oxygen aging, thermal oxidation aging occurs during asphalt pavement construction including asphalt mixture mixing, laying and compaction. Thermal oxidation aging is mainly due to thermal oxidation and light component loss of asphalt binders at high temperatures [2,3]. Thermal oxidation aging is usually simulated using the rolling thin film oven test (RTFOT) based on ASTM D2872-04 and pressure aging vessel test (PAV) based on ASTM D6521. For photo-oxygen aging, as early as 1961, Traxler found that light could accelerate the aging of asphalt pavement materials [4].

Further study showed that the ultraviolet radiation will break the energy-rich bond, such as C-C and C-H, of the polymer chain in the asphalt. A series of photo-oxidative reactions will occur, thus changing the composition and structure of the asphalt and deteriorating its mechanical properties [5,6]. It is general to adopt the ultraviolet lamp or the high-pressure mercury lamp as the light source. In addition, the xenon lamp is also used as the light source because its spectral lines are similar with those of sunlight [7,8]. The radiation intensity and light duration are generally set according to the actual solar radiation in the target region. For water-damage aging, indoor simulation of water damage aging methods include boiling method, immersion method, and freezing and thawing cycle method. By these methods, researchers found that water damage of asphalt binder mainly exists in two aspects. One is that the water diffuses and permeates in the asphalt binder, and reacts with water-sensitive components and polar groups. This leads to the gradual deterioration of the asphalt [9,10,11]. The other is that the deterioration of the asphalt performance attenuates the adhesion between the binder and the aggregates [12,13,14,15].

However, the long-term effects of light, oxygen, humidity and heat jointly lead to the aging of asphalt pavement materials and degradation of pavement performance. In order to simulate the actual long-term aging process of asphalt pavement materials, it is necessary to consider the multi-factor coupling aging test of asphalt and asphalt mixture, as the climate aging mode based on the actual environment.

Additionally, asphalt mastic is a kind of mixture of asphalt and mineral powder with a particle size less than 0.075 mm, which binds coarse and fine aggregates into a whole, so it plays a crucial role in the macroscopic properties of asphalt concrete (such as viscoelasticity and fatigue) [16]. In the early years, based on the dynamic shear rheometer, the fatigue factor of asphalt binder at 1% strain level and 10 Hz loading frequency was used as the evaluation index of asphalt fatigue performance [17]. Later, time scanning tests for controlling stress or strain were widely used [18]. In recent years, Johnson et al. proposed linear amplitude scanning test (LAS) for asphalt cement based on viscoelastic continuous damage theory (VECD) to solve the defect of time-consuming time scanning test [19,20]. Due to the differences between asphalt and asphalt mastic, specific test scheme setting and evaluation index selection of asphalt mortar still need to be modified.

Therefore, based on the specific climate of northeast China, the long-term effects of multi-factors, such as sun-light, oxygen, and water were considered comprehensively. The base asphalt mastic and SBS modified asphalt mastic were selected as the object, which were made of limestone powder mixed with 70# base asphalt and SBS modified asphalt, respectively. An indoor accelerated weather aging test method was designed, which could simulate the environmental conditions of the seasonal frozen region in northeast China. The viscoelasticity and fatigue performance of asphalt mastic with different aging degrees were studied. It is attempted to provide theoretical and experimental basis for optimizing the selection of asphalt pavement materials and the determination of filler–asphalt ratio in the seasonal frozen region of northeast China.

## 2. Experiment

### 2.1. Raw Materials

The raw material used in this paper was Pen 70 matrix asphalt produced by Inner Mongolia Luant Asphalt Hi-Tech Co.,Wuxi, China SBS modified asphalt was the commercial product with PG70-28 that supplied by Hubei Guochuang Hi-Tech Materials Co, Wuhan, China. In accordance with the testing methods in the “Standard Test Methods of Bitumen and Bituminous Mixtures for Highway Engineering” (JTG E20-2011), several technical indicators of base asphalt, modified asphalt and limestone powder were tested. The testing results are shown in Table 1 and Table 2. All the indicators meet the technical requirements in the “Technical Specification for Construction of Highway Asphalt Pavements” (JTG F40-2004). Limestone powder was obtained by grinding Guilin limestone finely with a density of 2.750 g/cm^3^ and a specific surface area of 0.582. X-ray fluorescence spectroscopy (Quant X type, Thermo Fisher Scientific Inc, Massachusetts, USA.) was used to analyze the raw material raw samples of limestone powder for compositional analysis, and the results are shown in the Table 3.

### 2.2. Preparation of Asphalt Mastic

**Figure 1:**
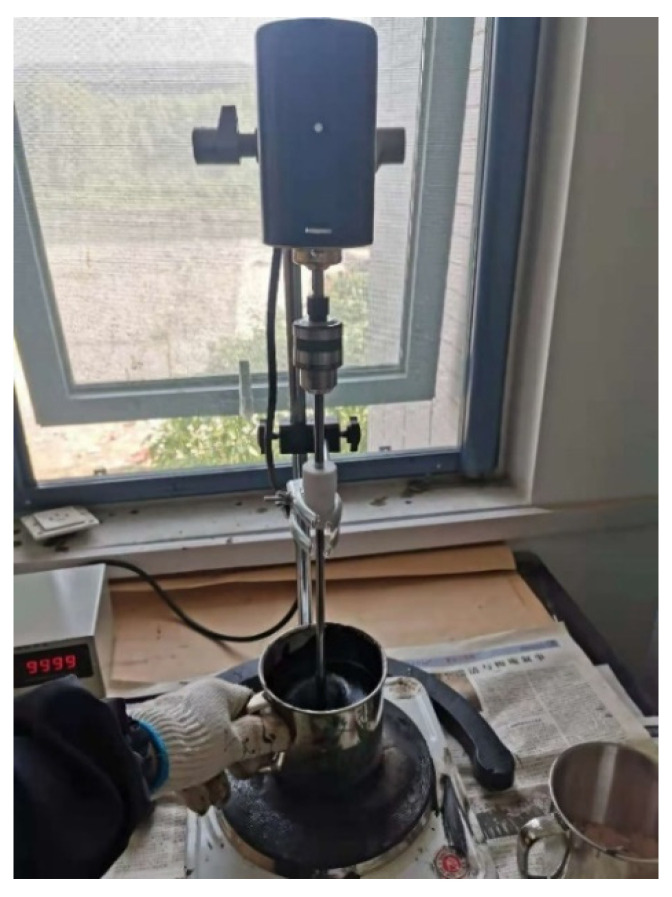
Preparation of asphalt mastic.

Five kinds of base asphalt mastic and SBS modified asphalt mastic were prepared with the filler-asphalt ratio of 0.6, 0.8, 1.0, 1.2, and 1.4. The preparation process was as follows:

(1) The asphalt was heated to a fluid state in the oven. The heating temperature of the base asphalt and SBS modified asphalt is 140 °C and 170 °C, respectively. A certain quality of the asphalt required for further tests was poured into the stainless steel cups;
(2) The limestone powder was heated in the oven at 140 °C for 2 h to make itself dry and the internal temperature uniform. The required quality of the limestone powder depended on the asphalt quality and the filler–asphalt ratio;
(3) The stainless steel cups containing the asphalt were heated on the electric stove to keep the asphalt in a fluid state during the mixing process. The mixing temperature was maintained at 140 °C ± 5 °C (base asphalt), 165 °C ± 5 °C (SBS modified asphalt);
(4) The limestone powder was added to the asphalt in batches, and mixed with the high-speed shearing machine. The speed was controlled at 1000 r/min and each mixing time was 3 min. After all the limestone powder was added, the asphalt mastic was mixed for another 5 min to ensure the even distribution of the limestone powder in the mixture, as shown in Figure 1;
(5) The evenly mixed asphalt mastic was poured into the silastic mold, making two kinds of samples with a thickness of 2 mm, a diameter of 8 mm and a thickness of 1 mm, a diameter of 25 mm. The asphalt mastic was also poured into the weather aging tray with 50 g per tray for short-term aging and weather aging tests.

### 2.3. Weather Aging Methods

**Figure 2:**
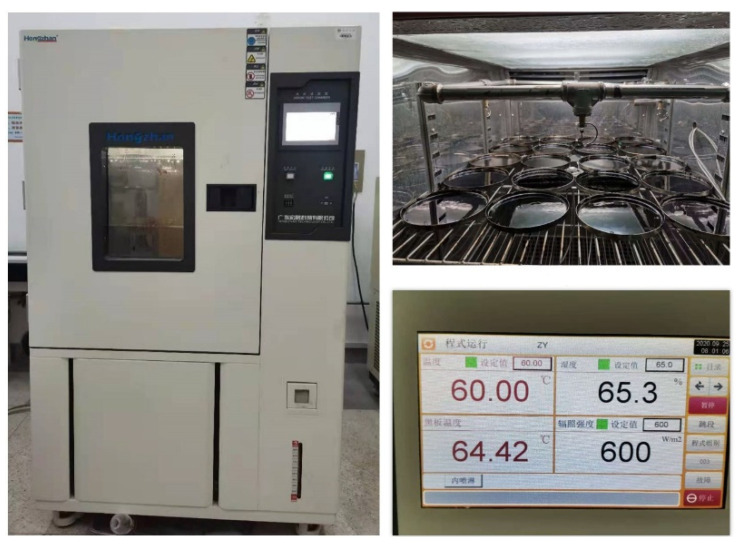
Weather aging apparatus and conditions.

In order to realize the aging effect of climate factors, such as sunlight, oxygen, temperature, and precipitation on asphalt mixture, this study adopted the weather aging box (Figure 2), referred to the artificial weathering aging method of vulcanized rubber in the standard GB/T 15255-2015 [21] and the accelerated aging test method for asphalt in ASTM D4798 [22], and comprehensively considered the natural environmental conditions in the seasonal frozen region of Northeast China. The conditions of the indoor accelerated weather aging test were as follows:

(1) The irradiation intensity of the xenon lamp was 600 W;
(2) The temperature of the black sheet was (65 ± 2.5) °C;
(3) The relative humidity was controlled at (65 ± 5) %;
(4) The rainfall intensity was 25 mm/h.

An aging cycle was 1 h, including 54 min of light and 6 min of rainfall. Moreover, the accelerated rate of asphalt aging was determined to be 3.84 by using a UV intensity meter to measure the UV radiation intensity of 135.6 W/m^2^ outdoors and 520 W/m^2^ inside the aging chamber, respectively. Meanwhile, taking into account the effects of water, temperature and airflow rate in the experiment, a correction factor 1.25 times was chosen to correct the accelerated rate of asphalt aging to 4.8. The average annual sunshine in the north-eastern region is 485.5 h. Combining this with the accelerated rate, it was found that 100 h of simulated aging in the laboratory is equivalent to 1 year of filed aging of the asphalt, and in this experiment the asphalt was aged for 100, 200, and 300 h to characterizes 1, 2, and 3 years of aging, and the specimens were turned over every 10 h.

**Figure 3:**
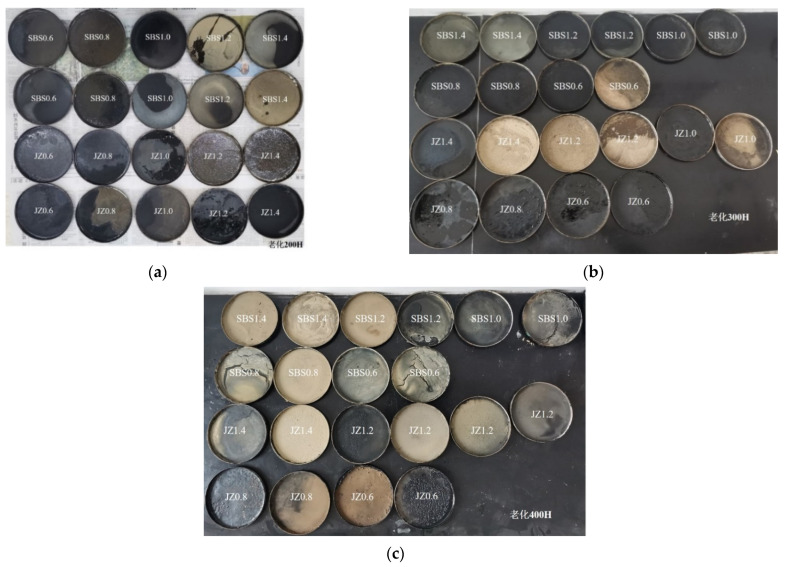
Asphalt mastic with different aging time. (**a**) aging for 200 h, (**b**) aging for 300 h, (**c**) aging for 400 h.

The asphalt mastic with different weather aging time was shown in Figure 3. It could be seen that as the aging time increased, a small amount of mineral powder precipitated in the asphalt mastic with a high filler–asphalt ratio. Generally, the precipitation phenomenon in the base asphalt mastic was more obvious. It means that in the environment of light, oxygen, and water, the stability of the asphalt mastic is affected by the content of the mineral powder. With the increase in the filler–asphalt ratio, the stability becomes worse.

### 2.4. Dynamic Frequency Sweep Test

**Figure 4:**
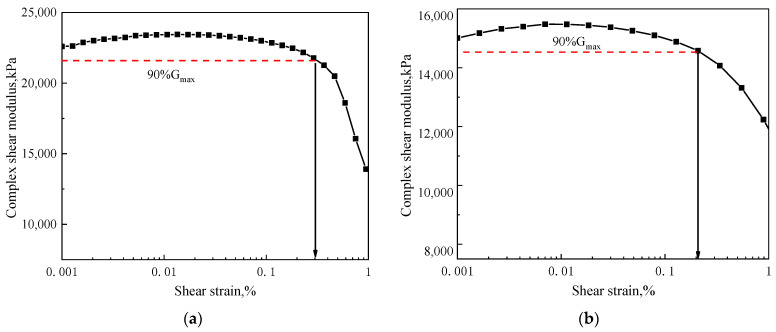
Strain sweep curves of asphalt mastic. (**a**) base asphalt mastic, (**b**) SBS modified asphalt mastic.

When the asphalt mastic is in the range of linear viscoelasticity, the stress–strain relationship is independent of the test temperature and loading frequency. The master curves of the viscoelastic parameters can be determined according to the time–temperature equivalence principle. Although in the range of non-linear viscoelasticity, the principle is no longer applicable. So, the dynamic rheological tests of asphalt mastic should be carried out within the range of linear viscoelasticity. Firstly, strain sweep is performed on the asphalt mastic to determine the linear viscoelasticity range. With the increase in the mineral powder content in the asphalt mastic, the structural asphalt increases, and the thickness of the asphalt film on the surface of the mineral powder decreases. The volume filling and hardening effect of the mineral powder on the asphalt mastic is enhanced, which macroscopically shows that the linear viscoelasticity range of the asphalt mastic decreases [23]. Meanwhile, the effect of weather aging will harden the asphalt mastic and reduce the linear viscoelasticity range. Therefore, base asphalt mastic and SBS modified asphalt mastic with the filler–asphalt ratio of 1.4 and aged for 400 h were selected for the strain sweep test. Figure 4 showed the sweep results of base asphalt mastic and SBS modified asphalt mastic at 20 °C and 10 Hz. According to the SHRP definition of the ultimate linear viscoelastic strain of asphalt materials, the linear viscoelastic limit is the strain at which the maximum complex shear modulus reduces to 90%. As shown in Figure 4, the complex shear modulus of base and modified asphalt mastic reduce to 90%Gmax were at 0.12% and 0.11%, respectively. Therefore, the linear viscoelastic strain range of the base asphalt mastic is 0–0.12% under the test conditions of 20 °C and 10 Hz, and that of the SBS modified asphalt mastic is 0–0.11%.

Based on the linear viscoelasticity range determined by the strain sweep test, the strain level of the frequency sweep test is set to 0.01%, and the loading frequency is 0.2 Hz, 0.4 Hz, 0.6 Hz, 0.8 Hz, 1 Hz, 2 Hz, 4 Hz, 6 Hz, 8 Hz, 10 Hz, 20 Hz, and 30 Hz. The test temperature is 5 °C, 15 °C, 25 °C, and 35 °C. Within the range of 10 °C to 30 °C, samples with a diameter of 8 mm and a height of 2 mm are used. When the test temperature is above 30 °C, samples with a diameter of 25 mm and a height of 1 mm are used. In order to analyze the viscoelastic characteristics of the asphalt mastic in a wider range of temperature and frequency, the WLF equation (Equation (1)) is adopted on the basis of the time–temperature equivalence principle. Taking 20 °C as the reference temperature, the shift factor is calculated. The complex shear modulus and phase angle at other temperatures and frequencies are shifted to the reference curve. Thus, the master curves of the complex shear modulus and phase angle of the asphalt mastic are obtained.

(1) $$\log\alpha_{T}=-\frac{C_{1}\left(T-T_{0}\right)}{C_{2}+\left(T-T_{0}\right)}$$

where, $\alpha_{T}$ is the shift factor at the reference temperature; *T* is the test temperature; *T*_0_ is the reference temperature; *C*_1_ and *C*_2_ are the fitting parameters.

Referring to the existing studies, the Sigmoidal, CA, and CAM models have relatively high fitting accuracy to the master curves of the complex shear modulus of the asphalt mastic [21]. Due to the clear physical meaning, the CAM model is adopted to fit the master curve of complex shear modulus, as shown in Equation (2).

(2) $$\left|G^{*}\right|=G_{g}{\left[1+{\left(\frac{\omega_{c}}{\omega_{r}}\right)}^{v}\right]}^{-\frac{w}{v}}$$

where, *G_g_* is the glassy shear modulus; $\omega_{c}$ is the crossover frequency; $\omega_{r}$ is the reduced frequency; $v=\log\left(\frac{2}{R}\right)$, *R* is the ratio of *G_g_* and $\left|G^{*}\right|$ at the crossover frequency; v is the parameter determining the shape of master curve.

Since the deviation in the low frequency range is large when the CAM model is used to fit the phase angle master curve of the asphalt mastic, the double-logistic model (Equation (3)) is adopted to fit the master curve of the phase angle [24]. The shift factor is the same as that of the complex shear modulus master curve.

(3) $$\delta =\delta_{P}-\delta_{P}\cdot H\left(f_{red}-f_{P}\right)\cdot \left(1-e^{-\left(S_{R}\cdot \log\left(\frac{f_{red}}{f_{P}}\right)\right)}{}^{{}^{{}^{{}^{{}^{{}^{{}^{2}}}}}}}\right)+\delta_{L}\cdot H\left(f_{P}-f_{red}\right)\cdot \left(1-e^{-\left(S_{L}\cdot \log\left(\frac{f_{P}}{f_{red}}\right)\right)}{}^{{}^{{}^{{}^{{}^{{}^{{}^{2}}}}}}}\right)$$

where, $\delta_{P}$ is the plateau phase angle; $f_{red}$ is the reduced frequency; $f_{P}$ is the frequency at the plateau; $H\left(f_{red}-f_{P}\right)$ and $H\left(f_{P}-f_{red}\right)$ are two heaviside step functions; $S_{R}$ stands for the rise (or fall) on the right side and $\delta_{L}$ for the left side of plateau $\delta_{P}$.

### 2.5. Fatigue Test

A new test based on the theory of viscoelastic continuum damage is adopted. According to the “Estimating damage tolerance of asphalt binders using the linear amplitude sweep” (AASHTO TP101-14), the test consists of two parts. Firstly, frequency sweep is carried out on the asphalt mastic at the designed temperature. The strain is 0.01% and the sweep frequency is 0.2 Hz, 0.4 Hz, 0.6 Hz, 0.8 Hz, 1 Hz, 2 Hz, 4 Hz, 6 Hz, 8 Hz, 10 Hz, 20 Hz, and 30 Hz. Based on complex shear modulus and phase angle, the undamaged material parameter α in the further fatigue damage analysis is calculated. Secondly, at a loading frequency of 10 Hz, the asphalt mastic is swept with a linear increase in strain amplitude from 0.1% to 30% within 310 s. The linear amplitude sweep test at 15 °C was performed on the asphalt mastic. It was found that the adhesive failure occurred quickly between the asphalt mastic and the upper and lower rotors. Therefore, the temperature of the linear amplitude sweep test in this study is set to 20 °C.

## 3. S_VECD Model and Fatigue Failure Index

### 3.1. S-VECD Model

The establishment of the simplified viscoelastic continuum damage (S-VECD) model is based on three principles.

(1) Elastic-viscoelastic correspondence principle based on the pseudo strain. The viscoelastic problem can be simplified into the elastic problem with this principle;
(2) Work potential theory based on the continuous damage mechanics, and it can quantify the influence of internal microdamage on the macroscopic mechanical behavior;
(3) Time–temperature equivalence principle. It includes the influence of loading time and temperature on the physical and mechanical behavior of materials [25].

(1) Elastic-viscoelastic correspondence principle based on the pseudo strain;

The linear viscoelastic materials are dependent on time and temperature. The machinal response is not only related to the current load, but also the entire loading history. The constitutive relation of viscoelastic materials can be represented by Equations (4) and (5).

(4) $$\sigma =\int_{0}^{t}E\left(t-\tau \right)\frac{d\varepsilon }{d\tau }d\tau$$

(5) $$\varepsilon =\int_{0}^{t}D\left(t-\tau \right)\frac{d\sigma }{d\tau }d\tau$$

where, E(t) and D(t) are, respectively, the relaxation modulus and creep compliance of materials; τ is the integration variable.

The Schapery elastic–viscoelastic correspondence principle replaces the viscoelastic strain of materials with the pseudo strain. The viscoelastic problem is transformed into the elastic problem, as shown in Equation (6).

(6) $$\varepsilon^{R}=\frac{1}{E_{R}}\int_{0}^{t}E\left(t-\tau \right)\frac{d\varepsilon }{d\tau }d\tau$$

where, $\varepsilon^{R}$ is the pseudo strain; ε is the physical strain of materials; *E_R_* is the specific reference modulus.

The constitutive relation of viscoelastic materials based on the pseudo strain can be obtained by bringing Equation (4) into Equation (6), as shown in Formula (7). For viscoelastic materials, the modulus attenuation under load is due to the combined effect of viscoelastic behavior and internal damage. The constitutive relation based on the pseudo strain can separate the viscoelastic behavior and the damage, thus effectively analyzing the damage development of materials under load.


(7) $$\sigma =E_{R}\varepsilon^{R}$$

(2) Work potential theory based on the continuous damage mechanics;

Since it is difficult to define and quantify the microdamage inside materials, the continuous damage mechanics describes the damage evolution process of materials by reducing the effective stiffness resulted from the microstructure change. The VECD model adopts Schapery work potential theory to quantify the damage. It defines the damage as the internal state variable of materials, and quantitatively analyzes the fatigue damage process.

The work potential principle of viscoelastic materials is shown in Equation (8).

(8) $$W^{R}=f\left(\varepsilon^{R},S\right)$$

where, $W^{R}$ is the pseudo strain energy density; S is the internal state variable of materials characterizing the damage.

Equation (9) expresses the damage rate of materials.

(9) $$\frac{dS}{dt}={\left(-\frac{\partial W^{R}}{\partial S}\right)}^{\alpha }$$

where, α is the material parameter under undamaged state, $\alpha =\frac{1}{m}$; m is the fitted slope of composite shear modulus master curve of materials in linear viscoelasticity range.

The pseudo strain energy density of materials can be calculated by the following Equation (10).

(10) $$W^{R}=\frac{1}{2}C\left(S\right){\left(\varepsilon^{R}\right)}^{2}$$

where, *C*(*S*) is the pseudo modulus, which is the function of material damage parameter *S*. It can be presented by Equations (11)–(13).

(11) $$C\left(S\right)=\frac{\tau_{P}}{\varepsilon_{P}^{R}\times DMR}$$

(12) $$\varepsilon_{P}^{R}=\varepsilon_{P}\times {\left|G^{*}\right|}_{0}$$

(13) $$DMR={\left|G^{*}\right|}_{I}/{\left|G^{*}\right|}_{0}$$

where, $\tau_{P}$ is the peak shear stress in any loading cycle; $\varepsilon_{P}^{R}$ is peak pseudo shear strain in any loading cycle; dynamic modulus ratio (DMR) is the specimen variability compensation parameter, which generally needs to be limited with 0.9~1.1; ${\left|G^{*}\right|}_{0}$ is the dynamic modulus at the simulated temperature and frequency; ${\left|G^{*}\right|}_{I}$ is the initial dynamic modulus.

The expression of damage variable S can be derived from Equations (9)–(12).

(14) $$S=\overset{N}{\underset{i=1}{\sum }}{\left[\frac{DMR}{2}{\left(\varepsilon_{P}^{R}\right)}^{2}\left(C_{i-1}-C_{i}\right)\right]}^{\frac{\alpha }{1+\alpha }}{\left[t_{i}-t_{i-1}\right]}^{\frac{1}{1+\alpha }}$$

where, i is the time interval selected for damage calculation.

The characteristic curve formula of material damage can be obtained by using power function to fit the pseudo modulus C and damage variable S of materials, as shown in Equation (15).

(15) $$C=1-C_{1}{\left(S\right)}^{C_{2}}$$

where, $C_{1}$ and $C_{2}$ are the fitting parameters.

The relationship between the cycle number N and peak shear strain $\varepsilon_{P}$ can be obtained by combining Equations (9), (10), (11), (12), and (15).

(16) $$N=\frac{f\cdot 2^{\alpha }\cdot S^{1-\alpha C_{2}+\alpha }}{\left(1-\alpha C_{2}+\alpha \right){\left(C_{1}C_{2}\right)}^{\alpha }{\left({\left|G^{*}\right|}_{0}\cdot \varepsilon_{P}\right)}^{2\alpha }}$$

where, f is the loading frequency.

Substituting the cumulative damage variable $S_{f}$ at fatigue failure into Equation (16), the number of cycles at failure $N_{f}$ can be estimated by the Equation (17):

(17) $$N_{f}=\frac{f\cdot 2^{\alpha }\cdot S_{f}^{1-\alpha C_{2}+\alpha }}{\left(1-\alpha C_{2}+\alpha \right){\left(C_{1}C_{2}\right)}^{\alpha }{\left({\left|G^{*}\right|}_{0}\cdot \varepsilon_{P}\right)}^{2\alpha }}$$

### 3.2. Fatigue Failure Criterion of Asphalt Mastic

The fatigue failure of materials should be determined according to the internal damage distribution. Existing methods make it difficult to obtain the physical condition change of material interior during the fatigue process. In general, the fatigue failure point of materials is defined based on the phenomenological method or energy method. In the time sweep test of asphalt, the main measures determining the fatigue life are as follows.

(1) Cycle number when the dynamic modulus is reduced to 50% of the initial modulus (*N_f_*_50_);
(2) Cycle number at the peak of $\left|G^{*}\right|\times i$, which *i* is the loading times (*N_NM_*);
(3) Cycle number at the inflection point of the change rate of $\left|G^{*}\right|\cdot (N_{G^{*}})$;
(4) Cycle number at the peak of energy dissipation rate (*N_DER_*);
(5) The fatigue life is determined on the basis of the cumulative dissipation energy ratio (CDER). Specifically, the relationship between CDER and the loading cycle is linear at the beginning. When the relation curve between CDER and the loading cycle deviates from the straight line by 20%, the material is considered to have fatigue failure. The cycle number at this time is defined as the fatigue life.

**Figure 5:**
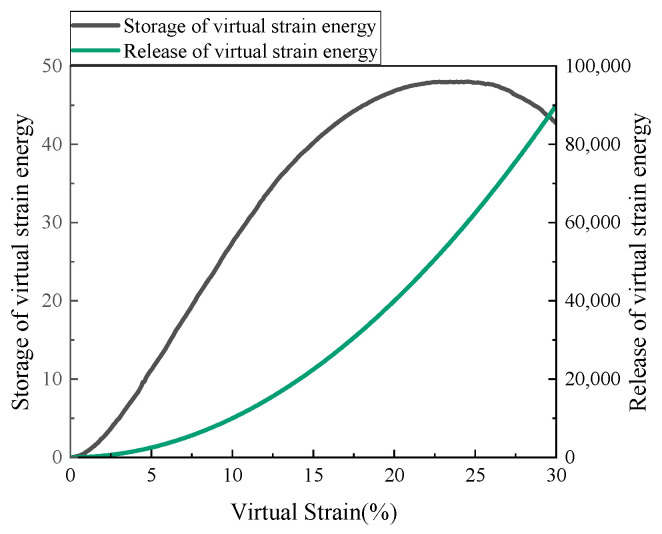
Curves of $W_{S}^{R}$ and $W_{r}^{R}$ in the LAS test.

In the researches on linear amplitude sweep (LAS) test of asphalt, a variety of measures can determine the material fatigue failure based on the phenomenological method or the energy method. AASHTO TP101 (2012) took the shear stress peak point as the fatigue failure point of asphalt. Although in the new version (2018), the fatigue failure was defined at the point when $\left|G^{*}\right|\sin\delta$ decreases to 65% of the initial value. Wang believed that although phenomenological indicators, such as peak shear stress and peak phase angle, can be used to define the fatigue failure of asphalt binders, it was necessary to establish a fatigue failure index based on the energy method, which could be used for VECD model prediction. The index related to pseudo strain energy was proposed [26]. Under the effect of load, the pseudo strain energy of viscoelastic materials $W_{t}^{R}$ includes the stored pseudo strain energy $W_{S}^{R}$ and released pseudo strain energy $W_{r}^{R}$, which are presented in Equations (17)–(20). Figure 5 shows the $W_{S}^{R}$ and $W_{r}^{R}$ curves of asphalt mastic in the LAS test. It can be seen that $W_{r}^{R}$ increases with the increase in the pseudo strain, and $W_{S}^{R}$ drops rapidly after reaching the peak. With the initiation and development of damage, the slope of $W_{r}^{R}$ curve increases gradually. It indicates that the energy storage capacity of materials decreases as the damage becomes worse. Therefore, the peak value of $W_{S}^{R}$ represents the maximum energy that the material can store, which can be used as the definition of asphalt fatigue failure. Recently, the fatigue failure criterion of asphalt binder based on $W_{S}^{R}$ has been applied to some extent, which proves its applicability in the asphalt fatigue behavior [27].

(18) $$W_{t}^{R}=\frac{1}{2}\cdot \tau_{undamaged}\cdot \varepsilon_{P}^{R}=\frac{1}{2}\cdot {\left(\varepsilon_{P}^{R}\right)}^{2}$$

(19) $$W_{S}^{R}=\frac{1}{2}\cdot \tau_{P}\cdot \varepsilon_{P}^{R}/DMR=\frac{1}{2}\cdot C\cdot {\left(\varepsilon_{P}^{R}\right)}^{2}$$

(20) $$W_{r}^{R}=W_{t}^{R}-W_{S}^{R}=\frac{1}{2}\cdot \left(1-C\right)\cdot {\left(\varepsilon_{P}^{R}\right)}^{2}$$

**Figure 6:**
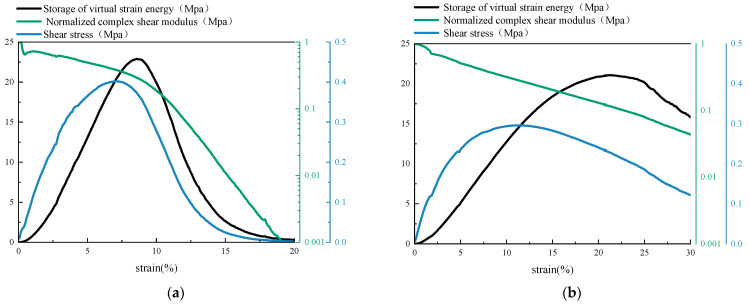
Curves of complex shear modulus, shear stress, and storage pseudo strain energy. (**a**) JZ0.6-O. (**b**) SBS0.6-O.

Figure 6 was the curves of complex shear modulus, shear stress, and storage pseudo strain energy for base asphalt mastic and SBS modified asphalt mastic. As could be seen, when the complex shear modulus dropped to 50% of the initial value, the shear stress, and stored pseudo strain energy of the asphalt mastic had not reached the peak value. It is conservative to use $0.5{\left|G^{*}\right|}_{0}$ as the fatigue failure index. If the inflection point of the complex shear modulus curve is taken as the fatigue failure point, the time at the inflection point of base asphalt mastic is basically the same as the time when $W_{S}^{R}$ reaches the maximum value (Figure 6a). Although, for SBS modified asphalt mastic, the complex shear modulus curve is still in a stable attenuation stage when $W_{S}^{R}$ reaches the maximum value (Figure 6b). The inflection point fails to occur by the end of the test. At this time, the attenuation amplitude of the shear stress curve is large and the obvious fatigue failure occurs. It is unreasonable to use the inflection point of the complex shear modulus curve as the fatigue failure index of SBS modified asphalt mastic. For two kinds of asphalt mastic, the peak of the shear stress occurs slightly earlier than that of $W_{S}^{R}$. It means that at the beginning of the shear stress attenuation, the energy storage capacity of the asphalt mastic is still in the enhancement stage. The material still has a strong carrying capacity. Therefore, compared with indexes related to shear stress and complex shear modulus, it is more suitable to use the maximum value of the stored pseudo strain energy to determine the fatigue failure of the asphalt mastic.

## 4. Results and Discussion

### 4.1. Influence of Weather Aging on Viscoelasticity Performance

**Figure 7:**
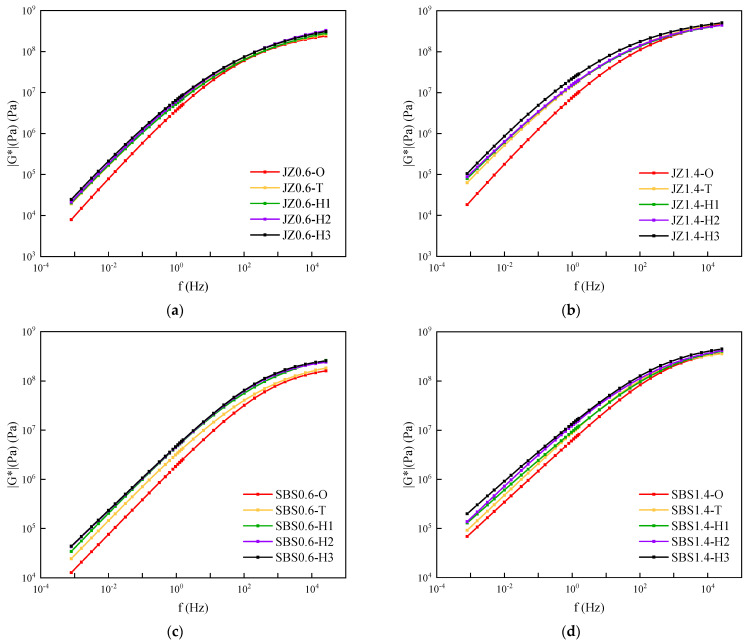
Complex shear modulus master curves of asphalt mastic with different aging degrees. (**a**) JZ0.6, (**b**) JZ1.4, (**c**) SBS0.6, (**d**) SBS1.4.

Taking the base asphalt mastic and SBS modified asphalt mastic with the filler–asphalt ratio of 0.6 and 1.4 as examples, the influence of weather aging on the viscoelasticity performance of the asphalt mastic was studied. Figure 7 showed the complex shear modulus master curves of asphalt mastic with different aging degrees. It was observed that with the increment of aging time, the complex shear modulus of the two kinds of asphalt mastic tended to increase. Under the condition of low-frequency loading, the complex shear modulus was sensitive to the effect of weather aging. The increment was significant. Although under high-frequency loading, the complex shear modulus tended to the same asymptotic line, less sensitive to weather aging. It is consist with the results of Liang et al., which studied the master curve of modified asphalt with different aging time and found [28]. The combined aging effect of light, oxygen and water increased the stiffness of the asphalt mastic. Due to the time limit of the aging test, the aging cycle interval was small. Thus, the variation of the complex shear modulus master curve with the aging time was relatively small.

**Figure 8:**
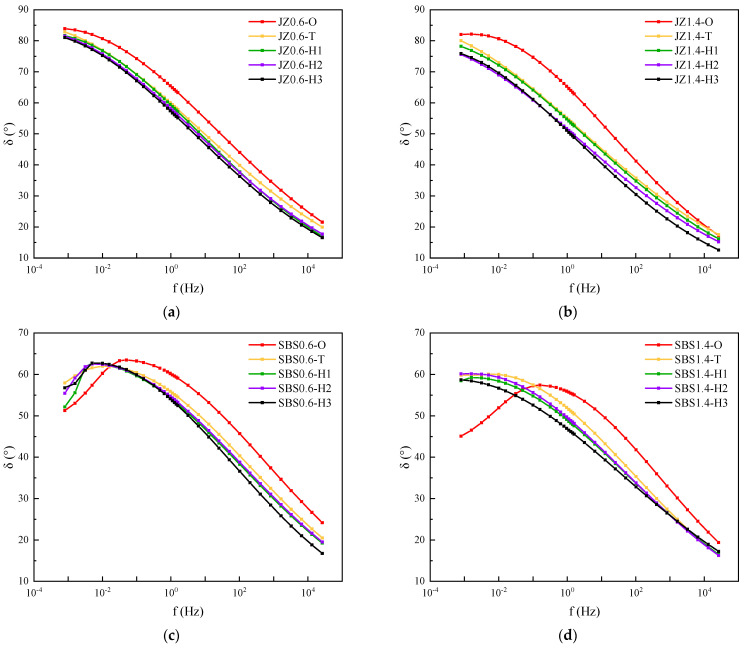
Phase angle master curves of asphalt mastic with different aging degrees. (**a**) JZ0.6, (**b**) JZ1.4, (**c**) SBS0.6, (**d**) SBS1.4.

Figure 8 was the phase angle master curve of base asphalt mastic and SBS modified asphalt mastic with different aging degrees. As the aging degree deepened, the phase angle of base asphalt mastic dropped. The elastic component increased while the viscous component decreased. For SBS modified asphalt mastic, the peak value of phase angle increased after short-term aging. The loading frequency corresponding to the peak phase angle decreased and the viscous component increased. In the weather aging stage, the peak value of phase angle dropped with the increment of aging time. According to the description of the aging mechanism of SBS modified bitumen, there are two parts of SBS modified bitumen aging, the degradation of the modifier and the change of the chemical composition of the asphalt [29]. Therefore, after conducted the short-term aging, SBS modifier was partially broken and decomposed into small molecules due to thermal oxidative aging and photo-oxidative aging. In this stage, the decomposition effect of SBS modifier is greater than the hardening effect caused by the oxidative dimerization of base asphalt. It increases the viscosity of SBS modified asphalt and the peak value of phase angle. During the stage of weather aging, with the continuity of thermal oxidative aging and photo-oxidative aging, the decomposition products and the small molecules in base asphalt polymerize to macromolecules. This gradually hardens SBS modified asphalt and decreases the phase angle. The elasticity of asphalt mastic increases and the viscosity drops.

### 4.2. Influence of Weather Aging on Fatigue Performance

The influence of weather aging on the fatigue performance of base asphalt mastic and SBS modified asphalt mastic was analyzed in this section. It should be noted that after aging for 400 h, the adhesive failure occurred quickly between the rotors and the base asphalt mastic with the filler–asphalt ratio of 1.2 and 1.4. This demonstrates that the adhesion performance of the base asphalt mastic with deep aging degree is reduced. The interface cracking is more likely to occur.

**Figure 9:**
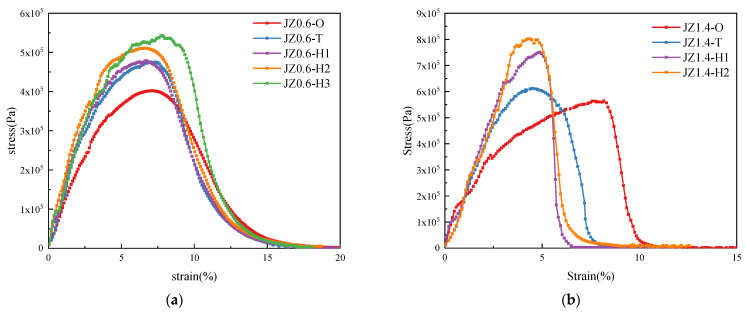

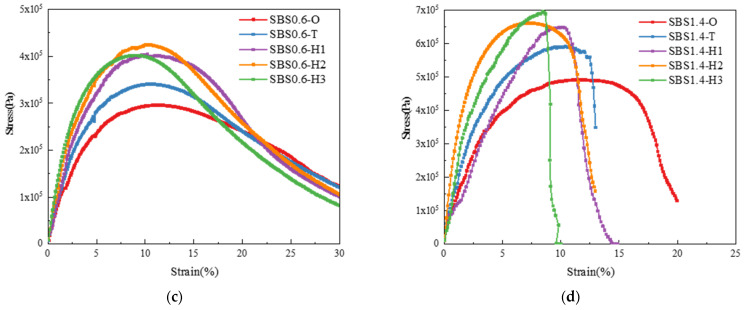
Stress–strain curves of asphalt mastic with different aging degrees. (**a**) JZ0.6. (**b**) JZ1.4. (**c**) SBS0.6. (**d**) SBS1.4.

The stress–strain curves of base asphalt mastic and SBS modified asphalt mastic with different aging degrees were presented in Figure 9. It showed that as the aging degree deepened, the peak shear stress of asphalt mastic increased. Comparing Figure 9a,b and Figure 9c,d, it could be seen that for the asphalt mastic with different mineral powder content, the shear strain corresponding to the peak shear stress varied differently with the aging degree. When the filler–asphalt ratio of asphalt mastic was 0.6, the shear strain corresponding to the peak shear stress was basically the same under different aging degrees. The shear strain when the fatigue failure occurred was also consistent. It indicated that the weather aging had less influence on the deformability of the asphalt mastic with a low filler–asphalt ratio. When the filler-asphalt ratio was 1.4, the shear strain corresponding to the peak shear stress decreased gradually with the deepening of aging degree. As the aging degree deepened, the brittle failure characteristics became more obvious and the failure strain was smaller. So, the deformability of the asphalt mastic with a high filler–asphalt ratio was greatly affected by weather aging.

**Figure 10:**
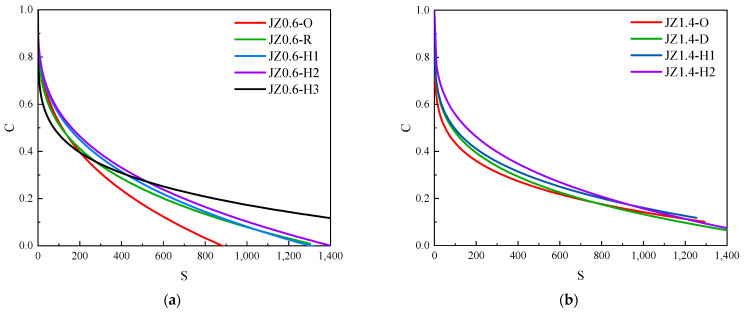

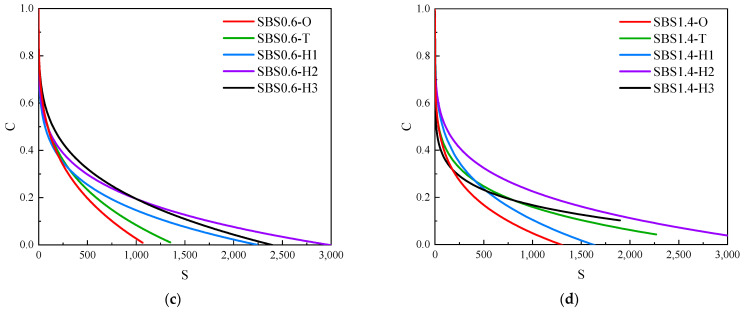
Curves of asphalt mastic with different aging degrees. (**a**) JZ0.6, (**b**) JZ1.4, (**c**) SBS0.6, (**d**) SBS1.4.

Figure 10 showed the *C* − *S* curves of base asphalt mastic and SBS modified asphalt mastic with different aging degrees. As can be seen in Figure 10a,b, within 300 h before weather aging, the *C* − *S* curves of base asphalt mastic with the filler–asphalt ratio of 0.6 and 1.4 rose gradually as the aging time increased. Under the same damage variable *S*, the material integrity of asphalt mastic was higher with the longer aging time. After 400 h of weather aging, the material integrity attenuation rate *C* of JZ0.6 asphalt mastic increased significantly in the initial damage state when *S* was less than 200. Once *S* was greater than 200, the attenuation rate *C* of JZ0.6 asphalt mastic was lower than that of asphalt mastic with low aging degree. In Figure 10c, within 300 h before weather aging, the *C* − *S* curves of SBS0.6 asphalt mastic showed a gradual rise trend. After 400 h of aging, the curves dropped in the late damage state. In Figure 10d, within 300 h before aging, the *C* − *S* curves of SBS1.4 asphalt mastic rose gradually with the increase in aging time. After being aged for 400 h, the curves dropped in the initial damage state when *S* was less than 250. The attenuation rate of material integrity increased. When *S* was greater than 250, the curves rose relatively and the material integrity attenuation rate decreased. It displayed significant damage resistance ability in the later stage.

**Figure 11:**
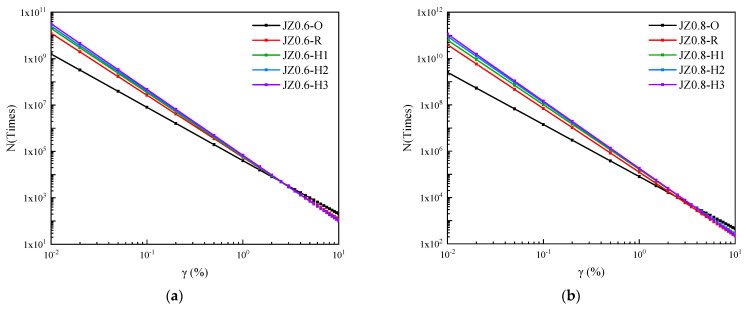

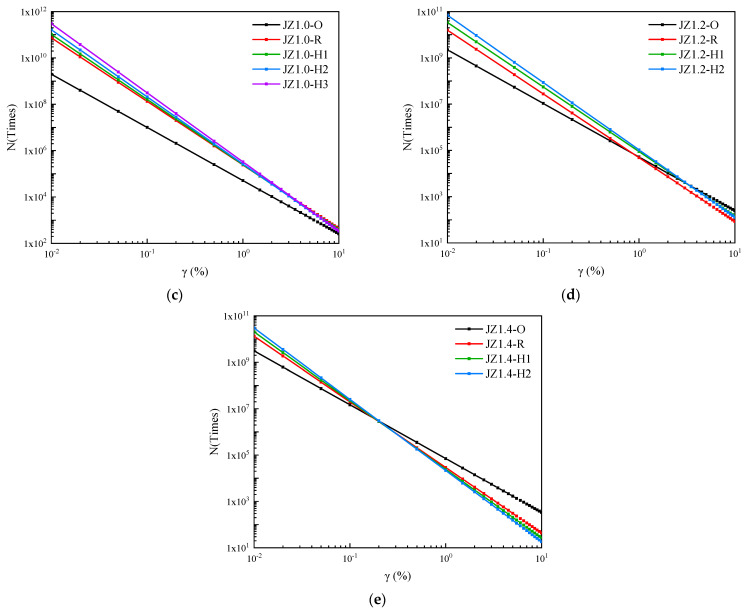
Fatigue life curves of base asphalt mastic with different aging degrees. (**a**) JZ0.6, (**b**) JZ0.8, (**c**) JZ1.0, (**d**) JZ1.2, (**e**) JZ1.4.

Figure 11 was the fatigue life curves of base asphalt mastic with different aging degrees. It presented that with the increment of strain level, the fatigue life attenuation rate of the base asphalt mastic was higher as the aging time became longer. The sensitivity to strain load became higher. The fatigue life of the aged base asphalt mastic increased under low strain load and decreased under high strain load. The fatigue life curves of base asphalt mastic with different aging degrees intersected in the middle strain interval. The strain corresponding to this intersection point is defined as the cross fatigue strain $\gamma_{c}$, which can be depicted in Equations (21)–(24).

The fatigue life Equation (17) can be represented in another way:

(21) $$N_{f}=A\gamma^{B}$$

where, $A=\frac{f\cdot 2^{\alpha }\cdot S_{f}^{1-\alpha C_{2}+\alpha }}{\left(1-\alpha C_{2}+\alpha \right){\left(C_{1}C_{2}\right)}^{\alpha }{\left({\left|G^{*}\right|}_{0}\right)}^{2\alpha }}$ and B=−2α.

The fatigue life equation at the undamaged interface is:

(22) $$N_{f_{0}}=A_{0}\gamma^{B_{0}}$$

The fatigue life equation at the damaged interface is:

(23) $$N_{f_{h}}=A_{h}\gamma^{B_{h}}\;$$

Let $N_{f_{h}}=N_{f_{0}}$, the calculation equation of the cross fatigue strain is obtained:

(24) $$\gamma_{c}={\left(\frac{A_{h}}{A_{0}}\right)}^{\frac{1}{B_{0}-B_{h}}}$$

When the strain load is $\gamma_{c}$, the fatigue life of the aged and unaged asphalt mastic is the same. When the strain load level is lower than $\gamma_{c}$, the fatigue life of the aged asphalt mastic is higher than that of the unaged asphalt mastic. It means that under low strain load, environment aging has a positive impact on the fatigue performance of asphalt mastic. When the strain load level is higher than $\gamma_{c}$, the fatigue life of the aged asphalt mastic is lower than that of the unaged asphalt mastic. It indicates that under high strain load, the influence of environmental aging on the fatigue performance of asphalt mastic is negative. According to the viscoelastic characteristic analysis of the asphalt mastic, the complex shear modulus of the aged asphalt mastic is relatively high. It shows high stiffness in terms of mechanical behavior. Under low strain load, the internal damage of the asphalt mastic is slight and the fatigue life is long due to the high strength. The deformation and bearing capacity of the aged asphalt mastic is relatively poor. The high strain load leads to the great internal damage of the asphalt mastic and the attenuation of fatigue life.

**Figure 12:**
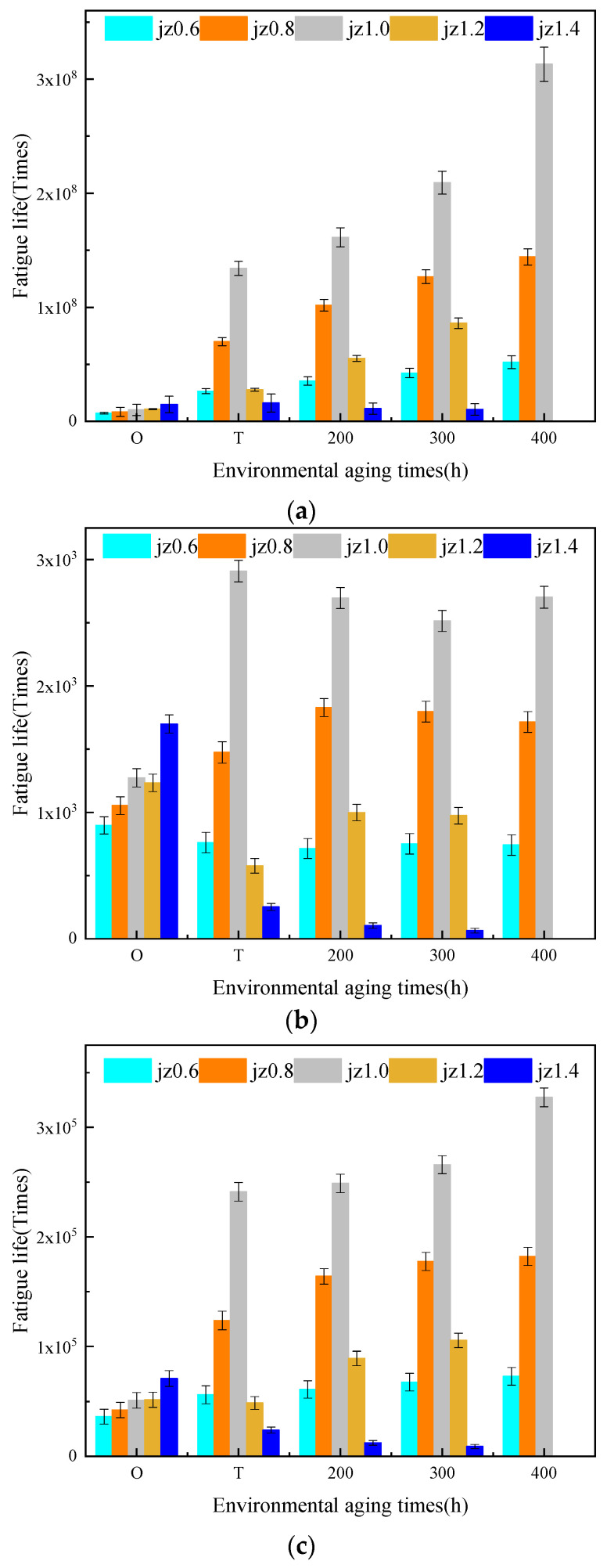
Fatigue life of base asphalt mastic with different aging degrees. (**a**) JZ-0.1%, (**b**) JZ-1%, (**c**) JZ-5%.

Table 4 summarized the $\gamma_{c}$ values of fatigue life curves for base asphalt mastic with different aging degrees. With the increment of the filler–asphalt ratio, $\gamma_{c}$ values of the aged and unaged asphalt mastic first increased and then decreased. The cross fatigue strain of the asphalt mastic with the filler-asphalt ratio of 0.6, 1.2, and 1.4 was relatively small. Under high strain load, weather aging had an adverse impact on the fatigue performance of these three kinds of asphalt mastic. The cross fatigue strain of the asphalt mastic with the filler–asphalt ratio of 0.8 and 1.0 was greater than 10%. Within the strain load range of 0.01% to 10%, the fatigue life of the two kinds of asphalt mastic increased after aging. The influence of weather aging on the fatigue life of the base asphalt mastic with middle filler–asphalt ratio was positive. Referring to Figure 12, the fatigue life of the unaged base asphalt mastic increased with the increase in filler–asphalt ratio. When the aging degree was the same, the aged base asphalt mastic with the filler–asphalt ratio of 1.0 had the maximum fatigue life under the strain load of 0.1%, 1%, and 5%. Therefore, based on the fatigue performance of the aged asphalt mastic, the best filler–asphalt ratio of the base asphalt mastic was 1.0.

**Figure 13:**
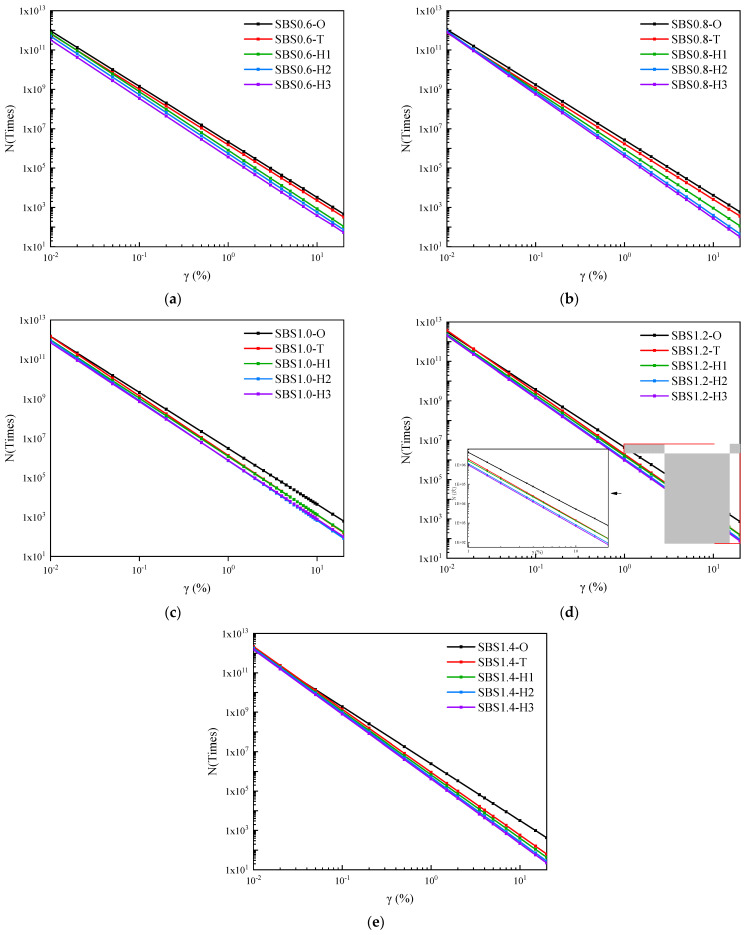
Fatigue life curves of SBS modified asphalt mastic with different aging degrees. (**a**) SBS0.6, (**b**) SBS0.8, (**c**) SBS1.0, (**d**) SBS1.2, (**e**) SBS1.4.

**Figure 14:**
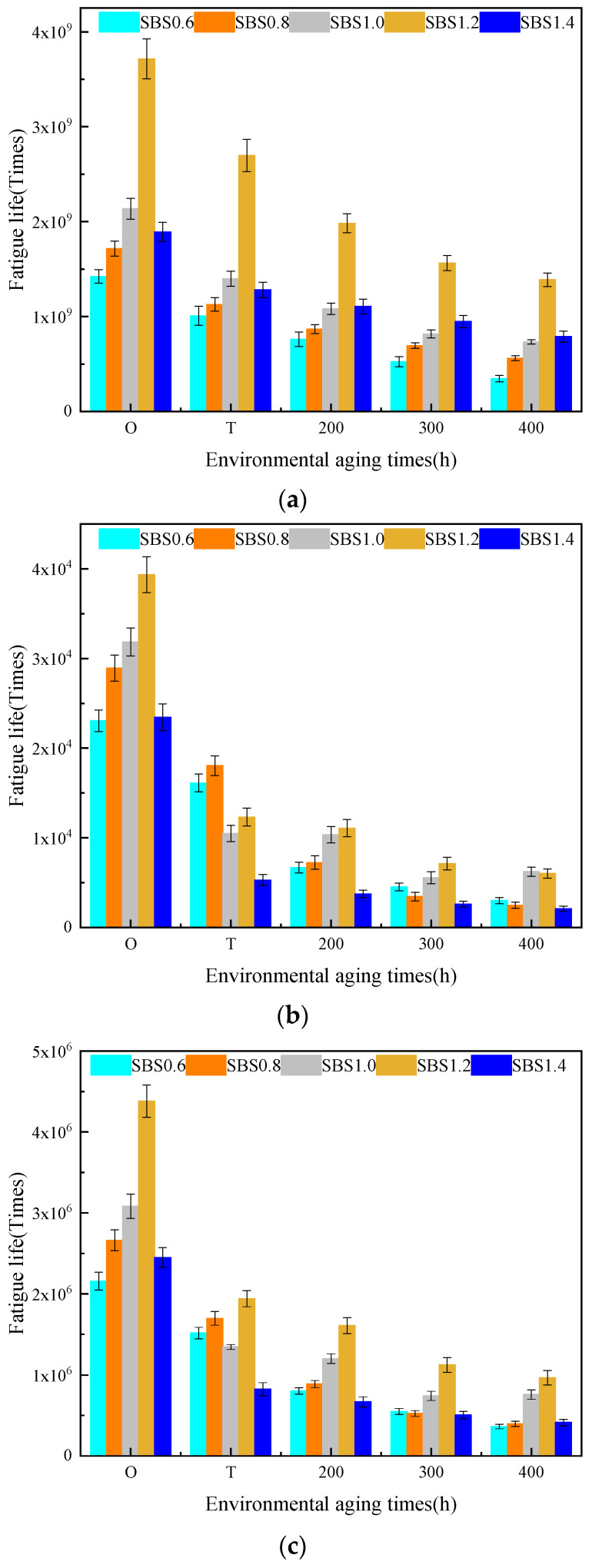
Fatigue life of SBS modified asphalt mastic with different aging degrees. (**a**) SBS-0.1%, (**b**) SBS-1%, (**c**) SBS-5%.

Figure 13 depicted the fatigue life of SBS modified asphalt mastic with different aging degrees. It pointed out that as the aging degree deepened, the fatigue life of SBS modified asphalt mastic was reduced within the strain load range of 0.01% to 10%. The slope of the fatigue life curves increased, which meant that the fatigue life became more sensitive to the strain load. Compared with base asphalt mastic, the influence of weather aging on the strain load sensitivity of SBS modified asphalt mastic was less. Within the strain load range, the cross fatigue strain failed to occur. Figure 14 presented the fatigue life of SBS modified asphalt mastic under the strain load of 0.1%, 0.5%, and 5%. When the filler–asphalt is less than 1.2, the fatigue life of asphalt mastic increased with the increase in the value of filler–asphalt ratio, and then reduced when the filler–asphalt ratio is 1.4. It indicated SBS modified asphalt mastic when the filler–asphalt ratio is 1.2 possesses the maximum fatigue life regardless of the degree of aging. Therefore, the fatigue performance of SBS modified asphalt mastic with different aging degrees was optimal when the filler–asphalt ratio was 1.2.

## 5. Conclusions

(1) Comparing the curves of complex shear modulus, shear stress, and storage pseudo strain energy for matrix asphalt mastic and SBS modified asphalt mastic, it is more suitable to use the maximum value of the stored pseudo strain energy to determine the fatigue failure of the asphalt mastic than shear stress and complex shear modulus;
(2) As the aging time increases, the complex shear modulus of asphalt mastic increases significantly under low-frequency loading. Although under high-frequency loading, the complex shear modulus tends to the same asymptotic line. With the deepening of aging degree, the phase angle of base asphalt mastic and SBS modified asphalt mastic drops. The elastic component increases while the viscous component decreases;
(3) According to the relationship between of fatigue life and the filler–asphalt ratio, the best filler–asphalt ratio of base asphalt and SBS modified asphalt are 1.0 and 1.2, respectively;
(4) The base asphalt mastic becomes more sensitive to the strain level after aging than SBS modified asphalt mastic. The fatigue life of base asphalt mastic increases under the low strain load and decreases under the high strain load, but the fatigue life of SBS modified asphalt mastic gradually decreases within the strain load range of 0.1% to 10% as the aging degree deepens.

## Figures and Tables

**Table 1:** Testing results of technical indicators for asphalt.

Technical Specifications		Base Asphalt	SBS Modified Asphalt
Penetration (25 °C, 100 g, 5 s) (0.1 mm)		69.3	54
Ductility (5 °C, 5 cm/min) (cm)		/	35
Ductility (10 °C, 5 cm/min) (cm)		43	/
Softening point (°C)		47	71.5
Thin film oven test TFOT (163 °C, 5 h)	Quality change (%)	0.1	0.1
	Residual penetration ratio (25 °C) (%)	65	76
	Residual ductility (5 °C) (cm)	/	22.6
	Residual ductility (10 °C) (cm)	26	/

**Table 2:** Testing results of technical indicators for mineral powder.

Test Item	Units	Testing Result
Apparent density	-	2.773
Hydrophilic coefficient	-	0.5
Plasticity	%	2.9
The water content	%	0.3
Appearance	-	No agglomeration

**Table 3:** Chemical composition of limestone powder.

Composition	LOSS	SiO_2_	Fe_2_O_3_	Al_2_O_3_	CaO	MgO	Cl^−^	K_2_O	SO_3_	CaCO_3_	Na_2_O
Percentage, %	41.00	4.47	0.47	0.85	50.70	0.87	0.024	0.48	0.17	91.50	0.07

**Table 4:** Cross fatigue strain of base asphalt mastic with different aging degrees.

Degree of Aging	Cross Fatigue Strain (%)				
	JZ0.6	JZ0.8	JZ1.0	JZ1.2	JZ1.4
T	3.23	10.41	31.17	0.88	0.11
H1	3.07	15.79	21.26	3.20	0.06
H2	3.50	12.98	15.47	3.35	0.06
H3	3.54	11.20	14.95	-	-

## Data Availability

Not applicable.

## References

[1] Feng Z.G., Bian H., Li X., Yu Y. (2016). FTIR analysis of UV aging on bitumen and its fractions. Mater. Struct..

[2] Poulikakos L.D., dos Santos S., Bueno M., Kuentzel S., Hugener M., Partl M.N. (2014). Influence of short and long term aging on chemical, microstructural and mac-ro-mechanical properties of recycled asphalt mixtures. Constr. Build. Mater..

[3] Wang M., Liu L. (2017). Investigation of microscale aging behavior of asphalt binders using atomic force microscopy. Constr. Build. Mater..

[4] Traxler R.N. (1961). Asphalt, Its Composition, Properties and Uses.

[5] Hu J., Wu S., Liu Q., Hernandez M.I.G., Wang Z., Nie S., Zhang G. (2018). Effect of ultraviolet radiation in different wavebands on bitumen. Constr. Build. Mater..

[6] Fernandez-Gomez W.D., Quintana H.A.R., Daza C.E., Lizcano F.A.R. (2014). The effects of environmental aging on Colombian asphalts. Fuel.

[7] Bai X., Qian G., Wei H., Jin D. (2019). Review on SBS Modified Asphalt Aging under Ultraviolet Light Radiation. J. Mater. Sci. Eng..

[8] Yu H.N., Yao D., Qian G.P., Cai J., Gong X.B., Cheng L.G. (2021). Effect of ultraviolet aging on dynamic mechanical properties of SBS modified asphalt mastic. Constr. Build. Mater..

[9] Noguera J.A.H., Quintana H.A.R., Gomez W.D.F. (2014). The influence of water on the oxidation of asphalt cements. Constr. Build. Mater..

[10] Pang L., Zhang X.M., Wu S.P., Ye Y., Li Y.Y. (2018). Influence of Water Solute Exposure on the Chemical Evolution and Rheolog-ical Properties of Asphalt. Materials.

[11] Xue-mei Z., Ling P., Guo-fu Z. (2018). Effects of the Aqueous Medium on Asphalt Aging Characteristic Functional. J. Wuhan Univ. Technol. (Transp. Sci. Eng.).

[12] Ji J., Suo Z., Wen B., Shi Y., Xu S. (2015). Effects of water and warm mix agent on adhesion capacity of ashalt-aggregate interface. China J. Highw. Transp..

[13] Liu X., Li B., Jia M., Li C., Zhang Z.W. (2020). Effect of short-term aging on interface-cracking behaviors of warm mix asphalt un-der dry and wet conditions. Constr. Build. Mater..

[14] Zheng C.F., Zheng S. Factors of Adhesion between Asphalt and Mineral Aggregates. Proceedings of the 2nd International Conference on Civil Engineering and Transportation (ICCET 2012).

[15] Sun W., Wang H. (2020). Moisture effect on nanostructure and adhesion energy of asphalt on aggregate surface: A molecular dy-namics study. Appl. Surf. Sci..

[16] Shashidhar N., Shenoy A. (2002). On using micromechanical models to describe dynamic mechanical behavior of asphalt mastics. Mech. Mater..

[17] Shenoy A. (2002). Fatigue testing and evaluation of asphalt binders using the dynamic shear rheometer. J. Test. Eval..

[18] Wang Y., Wang C., Bahia H. (2017). Comparison of the fatigue failure behaviour for asphalt binder using both cyclic and monotonic loading modes. Constr. Build. Mater..

[19] Xie W., Castorena C., Wang C., Kim Y.R. (2017). A framework to characterize the healing potential of asphalt binder using the linear amplitude sweep test. Constr. Build. Mater..

[20] Mannan U.A., Islam M.R., Tarefder R.A. (2015). Effects of recycled asphalt pavements on the fatigue life of asphalt under different strain levels and loading frequencies. Int. J. Fatigue.

[21] GB/T 15255-2015 (2015). Rubber, Vulcanized—Test Method for Artificial Weathering—Carbon-Arc Lamp.

[22] ASTM D4798/D4798M-11(2021) (2021). Standard Practice for Accelerated Weathering Test Conditions and Procedures for Bituminous Materials (Xenon-Arc Method).

[23] Li F., Yang Y.Y. (2021). Experimental investigation on the influence of interfacial effects of limestone and fly ash filler particles in asphalt binder on mastic aging behaviors. Constr. Build. Mater..

[24] Asgharzadeh S.M., Tabatabaee N., Naderi K., Partl M. (2013). An empirical model for modified bituminous binder master curves. Mater. Struct..

[25] Underwood B.S., Kim Y.R., Guddati M.N. (2010). Improved calculation method of damage parameter in viscoelastic continuum damage model. Int. J. Pavement Eng..

[26] Wang C., Castorena C., Zhang J.X., Kim Y.R. (2015). Unified failure criterion for asphalt binder under cyclic fatigue loading. Road Mater. Pavement Des..

[27] Zhang H.Y., Shen K.R., Xu G., Tong J.S., Wang R., Cai D.G., Chen X.H. (2020). Fatigue resistance of aged asphalt binders, An investigation of different analytical methods in linear amplitude sweep test. Constr. Build. Mater..

[28] Liang Y., Harvey J.T., Jones D., Wu R. (2021). Evaluation of Age-Hardening on Long-Term Aged Asphalt Binders. Constr. Build. Mater..

[29] Hu D., Gu X., Lyu L., Pei J., Cui B. (2021). Investigating the aging mechanism of asphaltene and its dependence on environmental factors through AIMD simulations and DFT calculations. Sci. Total Environ..

